# Response to Bollig and Zelco

**DOI:** 10.1186/s13049-025-01394-3

**Published:** 2025-05-01

**Authors:** Matthew Hooper, Marius Rehn

**Affiliations:** 1 MedSTAR Emergency Medical Retrieval Service. South Australian Ambulance Service, Adelaide, Australia; 2 Calvary Health Care. Adelaide, Adelaide, Australia; 3 School of Public Health and Tropical Medicine, James Cook University, Townsville, Australia; 4 Air Ambulance Department. Division of Prehospital Services, Oslo University Hospital, Oslo, Norway; 5 Institute of Clinical Medicine, University of Oslo, Oslo, Norway; 6 Department of Research and Development, Norwegian Air Ambulance Foundation, Oslo, Norway

## Abstract

In this reply we discuss the comments raised by Bollig and Zelco on our Editorial on Emergency Last Aid (ELA). We focus on that ELA is a clinician-focused educational initiative specifically tailored for those working in high-acuity, high-consequence settings such as pre-hospital, emergency, and critical care environments. The course is designed specifically for clinicians operating in environments where leadership, decision-making, and compassionate communication often occur under intense pressure, frequently with limited time and support.

## Main text

We thank Bollig and Zelco for their thoughtful response to our editorial on Emergency Last Aid (ELA) [1]. As we noted, the concept of “Last Aid” education is not new and has already made significant contributions to enhancing Public Palliative Care Education (PPCE). We referenced this work with respect, acknowledging its role in promoting death literacy and supporting non-medical caregivers.

However, we wish to clarify that the Emergency Last Aid (ELA) program we propose is distinct in both design and purpose. It is not derived from any existing, published Last Aid Course (LAC) framework and is not intended for a general audience. Rather, ELA is a clinician-focused educational initiative specifically tailored for those working in high-acuity, high-consequence settings such as pre-hospital, emergency, and critical care environments.

We note that while Bollig et al.’s published experience implementing LAC in hospital environments reported a 30.9% healthcare professional participation rate, only 1.8% (*N* = 1) of participants were physicians [2]. This underscores the very gap we aim to address: the absence of a formal, multi-disciplinary, and standardised educational framework for acute care clinicians who regularly manage the dying process under highly complex and emotionally charged conditions.

We appreciate the reference to the Last Aid Course for Professionals (LACP) program in Germany and look forward to reviewing its analysis when published. However, we reiterate that the ELA program we described is designed specifically for clinicians operating in environments where leadership, decision-making, and compassionate communication often occur under intense pressure, frequently with limited time and support.

To clarify another point raised: our reference to “leadership” in the editorial does not refer to administrative or organisational leadership, but to the human factors skills essential for guiding teams, patients, families, and bystanders through the chaos and trauma of death in acute settings. This includes leading resuscitation efforts that transition into end-of-life care, supporting distressed team members, and making ethically complex decisions in real time. These are core competencies in emergency and pre-hospital medicine and will be integral to the ELA framework.

The proposed ELA program will be developed and delivered by expert clinicians with frontline experience in pre-hospital, emergency, and critical care medicine. It aims to meet this group’s specific, unmet educational needs— currently not addressed by existing Last Aid models or palliative care curricula. Just as first aid laid the groundwork for the evolution of critical care, we believe ELA has the potential to transform how clinicians approach death, not as a failure, but as an essential moment of care deserving of the same skill, preparation, and compassion as any life-saving intervention.

We welcome further dialogue and collaboration with colleagues who are committed to advancing end-of-life care education across all domains of medicine.

## Data Availability

No datasets were generated or analysed during the current study.
